# Treatment Outcome of Neurogenic Bladder Dysfunction in Children; A Five-Year Experience

**Published:** 2014-06

**Authors:** Fathollah Roshanzamir, Mohsen Rouzrokh, Alireza Mirshemirani, Ahmad Khaleghnejad, Leila Mohajerzadeh, Reza Dalirani

**Affiliations:** 1 Pediatric Surgery Research Center; 2 Pediatric Nephrology Research Center, Shahid Beheshti University of Medical Sciences, Tehran, Iran

**Keywords:** Neurogenic Bladder;Treatment Outcome;Urinary Diverssion

## Abstract

***Objective:*** A neurogenic bladder is one which functions abnormally due to disorders of sacral nerves that control the bladder's ability to fill, store and empty urine. Abnormal bladder function can cause the bladder to be underactive or overactive. This study was planned to evaluate the treatment outcome of our patients with neurogenic bladder dysfunction (NGBD).

***Methods:*** Thirty three patients who have been treated for NGBD were evaluated. Diagnosis was confirmed by voiding-cysto-urethrography (VCUG) and urodynamic study. The patients were treated medically and all had clean intermittent catheterization (CIC). Data regarding age, sex, clinical and paraclinical findings, sonography, imagings, renal scan, associated anomalies, treatment and outcomes were collected and entered in SPSS software version18 and analyzed by descriptive statistical.

***Findings*** ***:*** Totally 33 patients aged three days to four years (mean 6.8 months) were included in this study. There were 20 (61%) males and 13 (39%) females. Mean follow-up period was 3.4±1.2 years (1.5 months to 5 years). Eighty two precent cases had bilatral and 18% unilatral hydronephrosis and bilatral vesicouretral reflux (VUR) existed in 67% and unilatral in 33% of the patients. Treatment consisted of antibiotherapy and CIC in all patients, which was only in 33% of the cases succesful. The most common associated anomaly was meningomyelocle in 8 patients. Vesicostomy was performed in 22 (67%) cases. Kidney scan showed scar in 10 patients at follow-up study. Complete continence on follow-up was achieved in 24 (71% ) patients, and it was improved in 6 (18% ) cases. Mortality rate was 9% (3 cases). Cure rate was 85% in urinary tract infection, 82.7% in hydronephrosis, 80% in VUR and 86.5% in kidney function.

***Conclusion:*** Anticholinergic medications was not effective in all our patients. We believe that permanent vesicostomy is an effective and acceptable surgical intervention for protection of upper urinary tract decompression, especially in those who do not respond to medical treatment and have high risk position.

## Introduction

Neurogenic bladder results from neurological lesions that end in a lower urinary tract dysfunction^[^^1^^]^, Abnormal bladder function can cause the bladder to be underactive (not emptying completely) or overactive (emptying too frequently/quickly). Children with NGB have a higher risk of urinary tract infection (UTI) and kidney damage. Neurogenic lower urinary tract dysfunction (NLUTD) may be caused by various diseases and events affecting the nervous system controlling the lower urinary tract. NLUTD depends grossly on the location and the extent of the neurologic lesion. Both in congenital and acquired NLUTD, early diagnosis and treatment is essential as irreversible changes may occur in paticular in children with myelomeningocle (MMC), but also in patients with traumatic spinal cord injury, even if the related neuropathologic signs were normal^[^^2^^,^^3^^]^. Diagnosis is based on history, physical examination, urodynamics, and typical manifestation of NLUTD. Treatment is based on non-invasive conservative measures such as catheterization, intravesical drug application (botulinum toxin injection, laser sphinctrotomy and urethral bulking agents). Surgical treatment can consist of urethral and bladder neck procedures, detrusor myectomy (auto-augmentation), denervation, covering bladder by striated muscle, bladder augmentation or substitution, and urinary diversion.

## Subjects and Methods

In a retrospective and descriptive study, 33 patients who have been treated for NGBD were evaluated in Mofid Children’s Hospital from January 2007 to December 2012. 

 Detailed history was taken and paraclinical examinations were performed and diagnosis was confirmed by ultrasonography (US), voiding-cysto-urethrography (VCUG), urodynamic study and lumbo-sacral MRI in myelodysplastic cases. We performed urodynamic evaluation without uroflowmetry in neonates after 3 months. 

 The patients were treated medically with anticholinergic and antibiotics and all had clean intermittent catheterization (CIC). Urine culture and sonography were checked every three months and diethylene triamine pentaacetic acid and dimercaptosuccinic acid renal scan every six months to one year, if necessary, during follow-up. Reduction in grade of hydronephrosis or vesicouretral reflux (VUR) was considered as improvement, and absence of disorders on evaluation was considered as cure. All records were evaluated and patients followed by personal visits in clinic or per phone call. Data regarding age, sex, clinical and paraclinical findings, sonography, imaging, renal scan, associated anomalies, treatment and outcomes were collected, entered in SPSS software version18 and analyzed by descriptive statistica.

**Table 1:** Age distribution of our patients

**Age group**	**Frequency (%)**
**<1 m**	8 (24.2%)
**1-12 m**	18 (54.6%)
**1-2 years**	5 (15.2%)
**2-4 years**	2 (6%)
**Total**	33 (100%)

***Findings***

Totally 33 patients aged three days to four years (mean 6.8 months) were included in this study (Table 1). There were 20 (61%) males and 13 (39%) females. Twenty five cases (76%) had UTI. Frequency and severity of hydronephrosis and VUR have been shown in Table 2. Blood urea nitrogen (BUN) in 4 patients (12.1%) and creatinine in 5 cases (15.2%) were normal. Four cases (12.1%) had creatinine more than 2 and 7 cases (21.2%) had BUN more than 30. 

 The most common associated anomaly was meningomyelocle in 8 patients (24.2%). Kidney anomaly, bladder anomaly, club foot and ureteropelvic junction obstruction were seen each one in 1 patient and 21 cases (63.6%) didn’t have any anomaly. Urodynamic study was performed in 20 cases (61%), and after three months in neonates without uroflowmetry, which confirmed NGB.

**Table 2:** Types and sites of hydronephrosis and vesicouretral reflux in our study group

**Complication**		**Mild**	**Moderate**	**Severe**
**Hydronephrosis**	**Bilatera**l	5	18	4
	**Unilateral (Rt:4**, **Lt:2)**	3	2	1
**Vesicouretral reflux**	**Bilateral**	6	9	7
	**Unilatera**l(**Rt:6, Lt:5**)	0	6	5

Rt: Right; Lt: Left

All patients received antibiotherapy and CIC, the treatment was succesful in 11 (33.3%) cases and 9 (27.3%) patients improved, but CIC was not successful in 13 cases (39.4%). 

 Diversion was performed in 22 (67%) cases who did not respond to medical treatment and were in high risk position for diversion. High levels of creatinine and BUN decreased to normal in 90% of patients after medical treatment, CIC and vesicostomy during follow-up. Kidney scan showed scar in 10 patients (30.3%) at follow-up study, 7 patients in left, 2 patients in right kinney and 1 patient bilatral. Complete continence on follow-up was achieved in 24 (71%) patients, and improved in 6 (18%) of cases. 9% (3 cases) died. Mean follow-up period was 3.4±1.2 years (1.5 months to 5 years). Cure rate was 85% in UTI (based on negative u/c), 82.7% in hydronephrosis (based on AP pelvic diameter), 80% in VUR (from grade V and IV to II and I), and 86.5% in kidney function (based on GFR/creatinine).

## Discussion

The diagnosis and treatment of NLUTD, which is a complex field, needs experience and requires up-to-date knowledge. The aims of treatment in NGB/NLUTD are the preservation of the upper urinary tract, bladder and bowel continence, independence, autonomy, and facilitation of self-estream^[^^4^^]^. After brief physical examination, urodynamic tests, uroflowmetry and ultrasound assessment are needed to clarify fine pathologic causes^[^^5^^]^. In cases with high detrusor pressure, the principal aim of treatment is conversion of high pressure bladder into a low-pressure reservoir. Alpha-blockers have been successful in decreasing bladder-outlet resistance, residual urine and automatic dysfunction^[^^6^^,^^7^^]^. Neurogenic bladder pathologies that commonly occur in patients with MMC include an elevated detrusor leak point pressure, VUR, and detrusor-external sphincter dyssynergia^[^^8^^-^^10^^]^. McGuire and colleagues^[^^11^^] ^first showed increased risk for upper tract dilatation in children with MMC. Shapiro and colleagues^[^^12^^]^ published outcomes of a 10-year therapy on 90 children with MMC treated with ileal loop diversion, and showed stable renal units in 69% of the patients. Kasabian and colleagues^[^^13^^] ^demonstrated normal renal function in 92% of children with MMC with voiding dysfunction treated with Oxybutynin and CIC. We peformed vicicostomy as a diversion In those patients who did not respond to medical treatment, were in risk position and had severe VUR and hydronephrosis., So we achieved a cure rate of 85% in UTI, 82.7% in hydronephrosis, 80% in VUR, and 86.5% in kidney function^[^^14^^,^^15^^]^. After failure of conservative treatment in patients with NGB urinary diversion represents a safe long-term compromise. In Stein et al^[^^16^^]^ study, upper urinary tract improved or remained stable in 97% of the renal units in patients with diversion, We performed too vesicostomy protection for urinary tract in some of our patients. Rawashdeh and colleagues^[^^17^^]^ in a retrospective study in children younger than 16 years old with NGB who had undergone detrusor myotomy showed a safe and effective alternative for the management of pharmacologically intratable NGBD in children. Murphy and colleagues^[^^18^^] ^reported obtaining total continency or major improvement with conservative care in 91% of 214 cerebral palsy patients with neurogenic bladder. Nue and colleagues^[^^19^^] ^evaluated the management of acquired NGB in children using intradetrusor botulinum toxin type A injection and achieved five (62.5%) patients completely dry. Complete continence on follow-up was achieved in 24 (71%) patients, and it was improved in 6 (18%) of cases in our study, but in Rawashdeh et al^[^^17^^]^ study it was reported in 8 (73%) patients, and improved only in one case. Stein et al^[^^16^^]^ reported 98% complete continence in those with a continent stoma. Murphy et al^[^^18^^]^ reported 91% total continence or major improvement with conservative care.

## Conclusion

Although anticholinergic medications and CIC have proved an effective treatment method for many children with NGB dysfunction, it was not effective in all our patients. We believe that permanent vesicostomy is an effective and acceptable surgical intervention for protection of upper urinary tract decompression, especially in those who do not respond to medical treatment and have high risk position.

## Authors’ Contribution

F. Roshanzamir: Acquisition of data, manuscript prepation and surgery.

M. Rouzrokh: Concept, design, data analysis and surgery.

A. Mirshemirani: Design, data analysis and surgery.

A. Khaleghnejad: Critical revision of manuscript, surgery and funds collection.

L. Mohajerzadeh: Data collection and analysis

R. Dalirani: Data collection, analysis and medical management.

All authors approved the final version of the manuscript.
